# Editorial: Changes in Molecular Expression After Memory Acquisition and Plasticity. Looking for the Memory Trace

**DOI:** 10.3389/fnmol.2020.00050

**Published:** 2020-04-03

**Authors:** Ramiro A. M. Freudenthal, Arturo Romano, Maria Veronica Baez

**Affiliations:** ^1^CONICET Institute of Physiology, Molecular Biology and Neurosciences (IFIBYNE), Buenos Aires, Argentina; ^2^Faculty of Exact and Natural Sciences, University of Buenos Aires, Buenos Aires, Argentina; ^3^CONICET Institute of Cell Biology and Neuroscience (IBCN), Buenos Aires, Argentina; ^4^Faculty of Medicine, University of Buenos Aires, Buenos Aires, Argentina

**Keywords:** Long Term Memory (LTM), molecular pathways, memory persistence, memory consolidation, molecular markers

Since Semon defined an engram as the neural substrate for memories (Semon, 1921, 1923), several hypotheses have been established, which had tried to determine how an engram is codified and where it is located in different brain structures. Historically, memory trace was defined as the physical mark of an acquired memory that in turn was considered a process divided in stages: acquisition, consolidation, and retrieval. The definition of the physical mark is central to understanding memory processing and helped to establish different scales at which these processes have been studied. Substantial support from research in several model systems points to the synaptic changes that occur initially during memory acquisition and consolidation and also during reconsolidation and extinction. Those changes, led to neurons to interact in a coordinate way that was called, neural assemblies. These assemblies are groups of cells that respond to specific stimulus in a coordinate way with a unique spatiotemporal pattern of firing. Based on that, it would be possible that neural assemblies would define the engram, as the physical mark of a memory (Nicolelis et al., 1997). Nevertheless, non-synaptic mechanisms have been described that are also capable of acting as memory substrates (Abraham et al., 2019). In this context, epigenetic intrinsic cellular adaptations emerge as a possible mechanism that regulate the pattern of RNA transcription and possibly indirectly other molecular dynamics that could initiate the engram at the intracellular level.

At present, we known that both, memories and the engram, are not fixed and inactive processes. Memory retrieval could turn the consolidated memory in a labile state, facilitating the update of the recalled memory in a process that was called reconsolidation. In parallel neural assemblies were modified in order to change the response based on the reconsolidation process.

In order to study where and how memory is codify and storage, and as an early approach, numerous authors performed lesions in different brain structures in order to establish which areas were related to the different memory stages (reviewed in Kandel et al., 2013). These works, established the first steps in the anatomical mapping of where memories were located.

In parallel, the study of patients such as H.M. confirmed the relationship between the hippocampus and the acquisition of new declarative memories and their consolidation mechanisms (Scoville and Milner, 1957; Milner, 1959).

On the other hand, the injection of several pharmacological agents helped to elucidate the neurotransmitters and post-synaptic receptors involved in memory processing (Kandel et al., 2013; Morris, 2013; Rossato et al., 2013). Also, it was discovered that, after memory acquisition, protein translation and gene expression were required, as well as protein acetylation, ubiquitination, and proteasome activity (Quevedo et al., 1999; Klann and Sweatt, 2008; Richter and Klann, 2009; Bousiges et al., 2010; Trinh and Klann, 2013; Jarome and Helmstetter, 2014). These processes take place along several time frames, starting immediately after memory acquisition and continuing during memory consolidation and subsequent phases (Quevedo et al., 1999; Jarome and Helmstetter, 2014; Figueiredo et al., 2015). More recently, epigenetic changes were incorporated to the engram hypothesis. DNA methylation and/or histone covalent modifications as acetylation, provides a different scale in gene transcription regulation as these mechanisms could enhance or silence different related genes simultaneously (D'Urso and Brickner, 2014; Lopez-Atalaya and Barco, 2014; Dean, 2017; Kim and Kaang, 2017). miRNAs also were associated to memory engram as they acts as post-transcriptional gene regulators in dendrite remodeling and synaptic plasticity, as well as in experience-dependent adaptive changes of neural circuits after memory acquisition (Bredy et al., 2011; Busto et al., 2015; Gu et al., 2015; Kremer et al., 2018). More recently, endogenous retrovirus were implicated in gene expression regulation associated to memory encoding, as they could share RNAs (mRNA, miRNA, circRNA) between cells modifying gene expression in connected cells (Hunter, 2019). A more interesting mechanism of Arc, that was classically related to memory encoding. It was recently discovery that Arc assembles itself as a retrovirus and move to other cells through the synapses trafficking information between neurons (Ashley et al., 2018; Pastuzyn et al., 2018).

In summary, many molecular changes are proposed as markers of the dynamic process that are part of the memory trace. More recently, *in vivo* electrophysiology and optogenetics have provided evidence that certain circuit changes are associated to memory encoding (Liu et al., 2012; Spiers and Bendor, 2014). However, as of today, more evidence is necessary to clarify how a new memory is storage in a circuit and how is it activated and could be modified during retrieval and reconsolidation.

The aim of this Research Topic is to discuss available data and to discover current developments in this area. Our goal is to join the latest advances in original research as well as updated reviews that contribute to the analysis and discussion of new ideas and recent discoveries in this area. Two papers dealing with this topic, Krawczyk et al. and Medina and Viola, discuss the relevance of the ERK1/2 pathway in Memory and Learning. In the first one, the authors review the role of ERK/MAPKs in reconsolidation of aversive and appetitive memories, highlighting the importance of ERK1/2 and Arc expression in the persistence of reactivated fear memories. In the second one, Medina and Viola discuss the role of the ERK1/2 pathway in each stage of the Memory process, and how these kinases link post-synaptic receptors with expression changes after memory acquisition.

In the same way, Pagani and Merlo debate about the differential role of kinases and phosphatases in Memory Formation and Extinction in associative long-term memories (LTM). Also, Zalcman et al. review the role of CAMKII isoforms in memory processing, emphasizing the importance of CAMKII α, β, γ, and δ isoforms and CAMKII heteromers formation in memory persistence.

Finally, we contribute original papers that investigate, on the one hand, the role of microRNA 210– 5p in cognitive impairment evidenced in a vascular dementia rat model induced by chronic cerebral ischemia (Ren et al.). On the other hand, Oliveira et al. study the strengthening of fear memories by epinephrine released during stressful events. Furthermore, Aguayo et al. analyze in review how transient changes in molecular synaptic composition that lead to memory consolidation are modified by stress exposure.

Taken together, data presented in this chapter look into several aspects of the elusive engram. In one hand, this topic highlights the role of kinases as ERK1/2 and CAMKII as well as phosphatases, as very important components of the engram, not only in the establishment of the trace, but also during reactivation and modifications of the engram. Furthermore, this chapter points recent findings that indicate that external stimulus could modify the engram components: enhancing, interfering, or deleting a memory trace.

## Author Contributions

MB wrote the editorial. AR and RF revised, corrected and made suggestions to the editorial.

### Conflict of Interest

The authors declare that the research was conducted in the absence of any commercial or financial relationships that could be construed as a potential conflict of interest.
